# Relationships among apical surface area, impaction level, and age in impacted mandibular third molars: a CBCT study

**DOI:** 10.1007/s11282-025-00868-5

**Published:** 2025-10-07

**Authors:** Ghassan Ali Abbas, Rabiah Al-Adawiyah Rahmat, Amir Hazwan Abdul Rahim, Meghna Gohain, Arofi Kurniawan, Mariam Abdullah, Zuraiza Mohamad Zaini, Norliza Ibrahim

**Affiliations:** 1 Department of Oral Diagnosis, Faculty of Dentistry, University of Karbala, Karbala, Iraq; 2 Department of Oral and Maxillofacial Clinical Sciences, Faculty of Dentistry, Universiti Malaya, Kuala Lumpur, Malaysia; 3 Department of Forensic Odontology, Faculty of Dental Medicine, Universitas Airlangga, Surabaya, Indonesia; 4 Department of Restorative Dentistry, Faculty of Dentistry, Universiti Malaya, Kuala Lumpur, Malaysia

**Keywords:** Age estimation, CBCT, Mandibular third molar, Tooth impaction, Young adult

## Abstract

**Objectives:**

This study investigated the association between the apical surface area measurement (ASAM) of impacted mandibular third molars (IMTM) and chronological age at varying impaction levels, as well as ASAM differences among levels.

**Methods:**

A total of 446 IMTM (227 right, 219 left) from 257 Malaysian patients aged 15.0 to 25.9 years were included and grouped into 0.9-year intervals. All CBCT images were analysed using Mimics and 3-matic software (Materialise NV, Belgium, version 21.0) to calculate the apical surface area. Impaction level was categorised according to the Pell and Gregory classification system. Spearman’s correlation and Welch’s ANOVA were conducted to assess the relationship between ASAM and age and to compare ASAM across the four levels (A, B, modified B/C, and C). Differences between left and right IMTM were assessed using an independent *t*-test, and analysed with SPSS (version 26).

**Result:**

ASAM showed an inverse correlation with age (*ρ* = −0.89, *ρ*^2^ = 0.79), with a median of 4.86 mm^2^ (IQR: 2.35–11.92). The steepest decline was observed between 17–18 years, followed by a plateau from 21 years onwards. Left and right IMTM were not statistically different (*p* = 0.53) and subsequently pooled. ASAM increased with impaction depth; however, only level C exhibited significantly larger ASAM than levels A and B (*p* < 0.001), with an effect size of *ω*^2^ = 0.13, indicating a notable delay in maturation.

**Conclusions:**

This study demonstrates the relationship between ASAM in IMTM and age, with potential applicability in dental age estimation. However, caution is advised for level C impactions, as the estimated age may be biased.

**Supplementary Information:**

The online version contains supplementary material available at 10.1007/s11282-025-00868-5.

## Introduction

The mandibular third molar (MTM) is the most commonly impacted tooth, with prevalence reported between 9.5 and 68% across populations [1, 2]. Impacted MTM (IMTM) are typically two-rooted (89.1%) but may present morphological complexities such as root curvature (40.7%), dilaceration (10%), and fusion (8.2%) [3]. In forensic and legal contexts, MTM is often used as a biological indicator for assessing whether an individual has attained the legal age of majority (18 years) [4], owing to the timing of its development and maturation [5]. However, studies have reported that impaction may be associated with delays in dental mineralisation ranging from 0.01 to 3.5 years [5–7], thereby compromising the accuracy of the estimated age and increasing the risk of bias. Consequently, because of both developmental delay and morphological complexity, IMTM are less favourable than their non-impacted counterparts for age estimation. Nevertheless, recent advances in three-dimensional (3D) imaging and improvements in image resolution, allow the precise visualisation of small and complex anatomical structures, thereby helping to mitigate some of these limitations.

The apical foramen of the MTM is one such structure that benefits from these imaging advances. As roots develop, the surface area of the apical foramen decreases in size until closure is reached, indicating dental maturity. Earlier age estimation methods studied this correlation with chronological age using two-dimensional (2D) imaging, and were subsequently refined using cone-beam computed tomography (CBCT) [8, 9]. Although strong correlations were reported and the resulting regression models showed reasonable accuracy, the possible influence of MTM impaction parameters was not adequately addressed.

One such parameter is the level or depth of impaction, typically assessed by comparing the occlusal plane of the MTM relative to the adjacent second molar [10]. To date, the influence of this parameter on apical maturation, in terms of surface area, and age, has not been investigated, although delayed development in impacted teeth is well documented. This study aimed to examine the relationship between the apical surface area measurement (ASAM) of IMTM and chronological age across different impaction levels, and to determine whether ASAM differs significantly between these levels.

## Materials and methods

### Materials

Ethical approval for the study was obtained from the Medical Ethics Committee (Ref. No: DF OS2103/0008(P)). This retrospective study was conducted in accordance with the principles of the Declaration of Helsinki and in accordance with local statutory requirements. Patients were informed that their CBCT images and data may be used for research and teaching purposes.

This was a cross-sectional study of 446 MTM from 257 CBCT images. The sample comprised 114 males and 143 females with bilateral or unilateral IMTM, aged 15.0–25.9 years. All CBCT scans were obtained using the i-CAT^™^ 3D Imaging System, version 3.1.62 (Imaging Sciences International Inc., Hatfield, PA, USA), between January 2008 and April 2019. Exposure parameters were 120 kVp, 18 mA, voxel size 300 µm, and a scanning time of 20 s. The field of view (FOV) was 13 cm × 17 cm and standardised for all images. The datasets were saved in Digital Imaging and Communications in Medicine (DICOM) format.

The inclusion criteria for the IMTM were healthy patients of Malaysian nationality, CBCT images with diagnostically acceptable quality, no metallic artifacts from adjacent regions, and pathology associated with the posterior mandible and/or the MTM. Developing MTM that had passed the average age of eruption but remained unerupted, as well as those with obstructed paths of eruption, were considered impacted and included. The sample size was calculated using G*Power software (version 3.1.9.4). F-test with a significant level of 0.02 (α = 0.02) and a power at 98% (1–β = 0.98). The effect size was equal to 0.06.

### 3D analysis of apical surface area

**Fig. 1:**
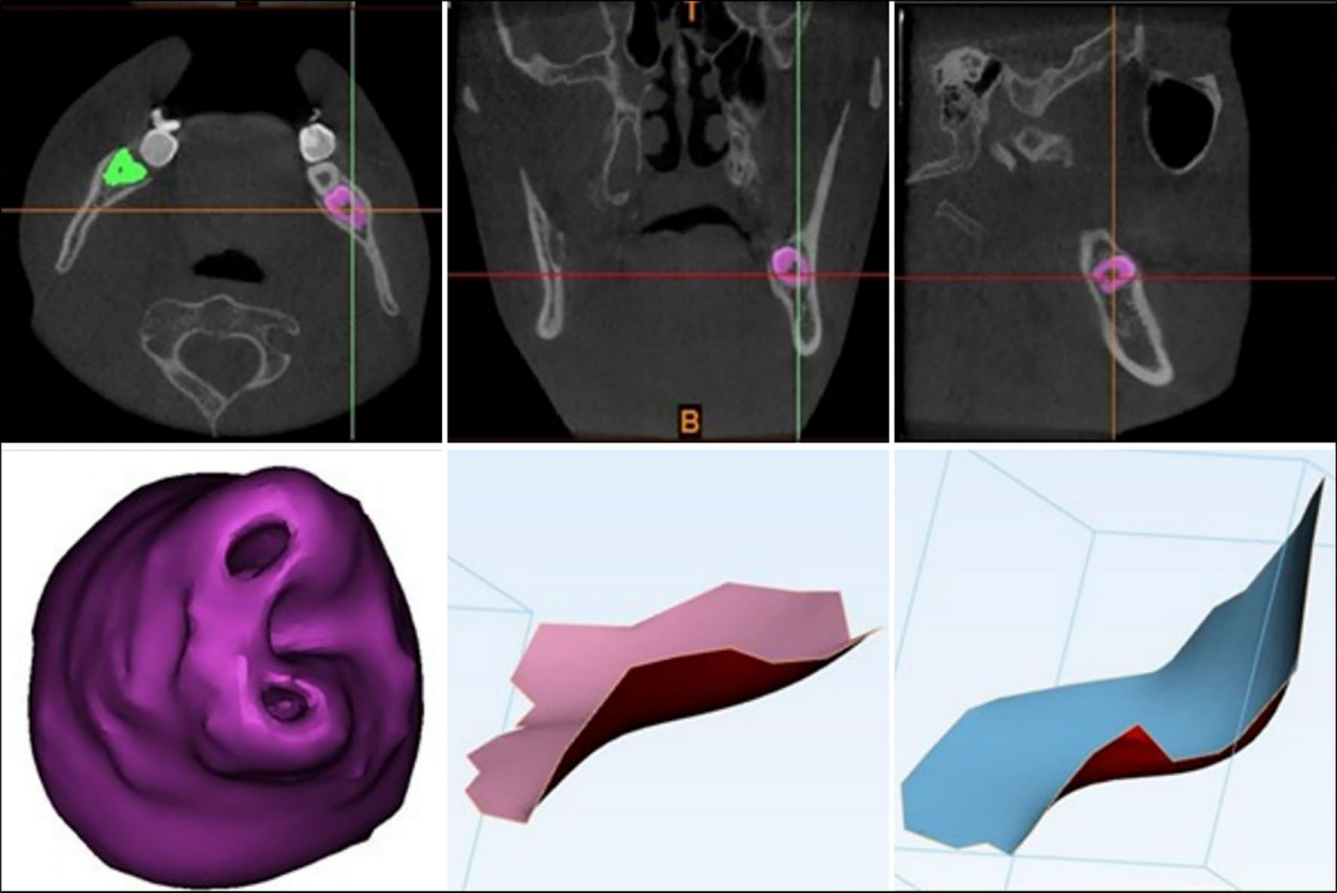
Multiplanar masking of IMTM for 3D reconstruction using Mimics software (top). The apical surface area was obtained from the reconstruction and calculated with 3-matic software (bottom)

The CBCT images were enhanced using Materialise Mimics (Materialise Interactive Medical Image Control System), a 3D image semi-automatic segmentation software (Materialise NV, Belgium, version 21.0) to generate the 3D models for IMTM. Figure 1 illustrates the process of creating new masks of IMTM by selecting predefined grayscale threshold values (1200–3071) after orienting the images in coronal, axial and sagittal views. The IMTM masks were manually cross-checked in the ‘multiple slice editing phase’ to allow accurate selection of calcified tooth structure, and to separate the tooth from the surrounding structures. The masks were then grown in the ‘region growing phase’ of the software. 3D models of IMTM were subsequently generated by the software. Smoothening effect of 0.6 was applied to select the margins of root apices.

The 3D models were then imported to 3-matic software (Materialise NV, Belgium, Version 13.0) for the surface area analysis. A ‘curve creation phase’ was used to create a curve along the margins of developing open apices. Based on the created curve, surface area was generated using ‘surface construction phase’. Finally, the surface area measurement was obtained. In cases of multi-rooted MTM, all apices were measured and the ASAM were averaged.

### Impaction level

**Fig. 2:**
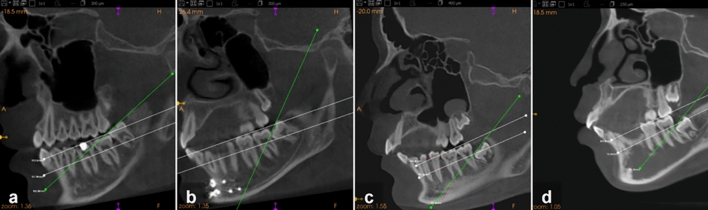
Sagittal CBCT images illustrating the four impaction levels. **a** Level A, **b** level B, **c** level B/C (modified) and **d** level C impaction. The green line indicates the occlusal plane of the IMTM and the white lines outline the occlusal plane and cervical margin of the adjacent mandibular second molar

The impaction levels were determined according to the IMTM occlusal level and classified using the Pell and Gregory system [10, 11] with Carestream 3D imaging software, as presented in Table 1 and illustrated in Fig. 2. A substantial number of IMTM with occlusal levels observed both above and below the cervical margin of the second molar, a variation not specified by the original authors. To address this, a modified fourth level, B/C, was introduced. The distribution of the sample by age group and impaction level is summarised in Table 2.

**Table 1:** Classification of impaction levels

Level	Description
A	The occlusal plane of the third molar is at the same level as the occlusal plane of the second molar
B	The occlusal plane of the third molar is between the occlusal plane and the cervical margin of the second molar
B/C	The occlusal plane of the third molar is evenly positioned both above and below the cervical margin of the second molar
C	The impacted tooth is below the cervical margin of the second molar

**Table 2:** Distribution of IMTM by age group (years) and impaction level

	A		B		B/C		C	
Group	N	Mean ± SD	N	Mean ± SD	N	Mean ± SD	N	Mean ± SD
15.0–15.9	–	–	12	15.4 ± 0.29	4	15.3 ± 0.11	22	15.2 ± 0.29
16.0–16.9	–	–	16	16.5 ± 0.17	–	–	13	16.4 ± 0.30
17.0–17.9	3	17.3 ± 0.03	11	17.6 ± 0.18	1	17.9	9	17.4 ± 0.29
18.0–18.9	–	–	26	18.3 ± 0.24	1	18.8	10	18.5 ± 0.30
19.0–19.9	1	19.8	41	19.6 ± 0.25	2	19.7 ± 0.11	9	19.6 ± 0.34
20.0–20.9	1	20.8	25	20.4 ± 0.33	4	20.6 ± 0.39	6	20.3 ± 0.34
21.0–21.9	4	21.5 ± 0.19	32	21.4 ± 0.27	1	21.9	9	21.7 ± 0.18
22.0–22.9	–	–	35	22.5 ± 0.32	2	22.5 ± 0.61	8	22.3 ± 0.20
23.0–23.9	1	23.6	44	23.5 ± 0.26	3	23.3 ± 0.16	14	23.5 ± 0.26
24.0–24.9	1	24.1	31	24.5 ± 0.23	–	–	9	24.4 ± 0.25
25.0–25.9	2	25.5 ± 0.50	27	25.5 ± 0.31	2	25.7 ± 0.28	4	25.3 ± 0.44
Total	13	21.3 ± 2.89	300	21.3 ± 2.81	20	20.4 ± 3.32	113	19.6 ± 3.36

*N* = number of teeth, *SD* = standard deviation

### Statistical analysis

Data were recorded in Microsoft Excel (Version 18, Microsoft Corporation) and are provided in Online Resource 1. Patient IDs were anonymised, and CBCT images were excluded to maintain confidentiality. Statistical analysis was conducted using IBM SPSS Statistics version 26.0 (IBM Corp., 2019). Association between ASAM and age was analysed using Spearman’s *ρ* correlation test, and mean ASAM was compared across impaction levels using Welch’s ANOVA and Games-Howell post-hoc analyses. The statistical significance was assessed using an independent *t*-test to compare ASAM values between the left and right IMTM with a *p*–value lower than 0.05 considered significant.

Reliability of the apical surface area measurements (ASAM) was assessed using intraclass correlation coefficient (ICC) analysis. To evaluate intra-examiner reliability, ASAM of 96 IMTM from a separate set of CBCT images were remeasured by the principal investigator after a one-month interval. To evaluate inter-examiner reliability, the same images were independently measured by another calibrated examiner, and the results were compared.

## Results

### Intra- and inter-examiner reliability

The ICC values for intra-examiner (0.94) and inter-examiner (0.91) reliability demonstrated excellent agreement, indicating that ASAM measurements from CBCT images were highly consistent both within and between examiners.

### Apical surface area measurement

**Fig. 3:**
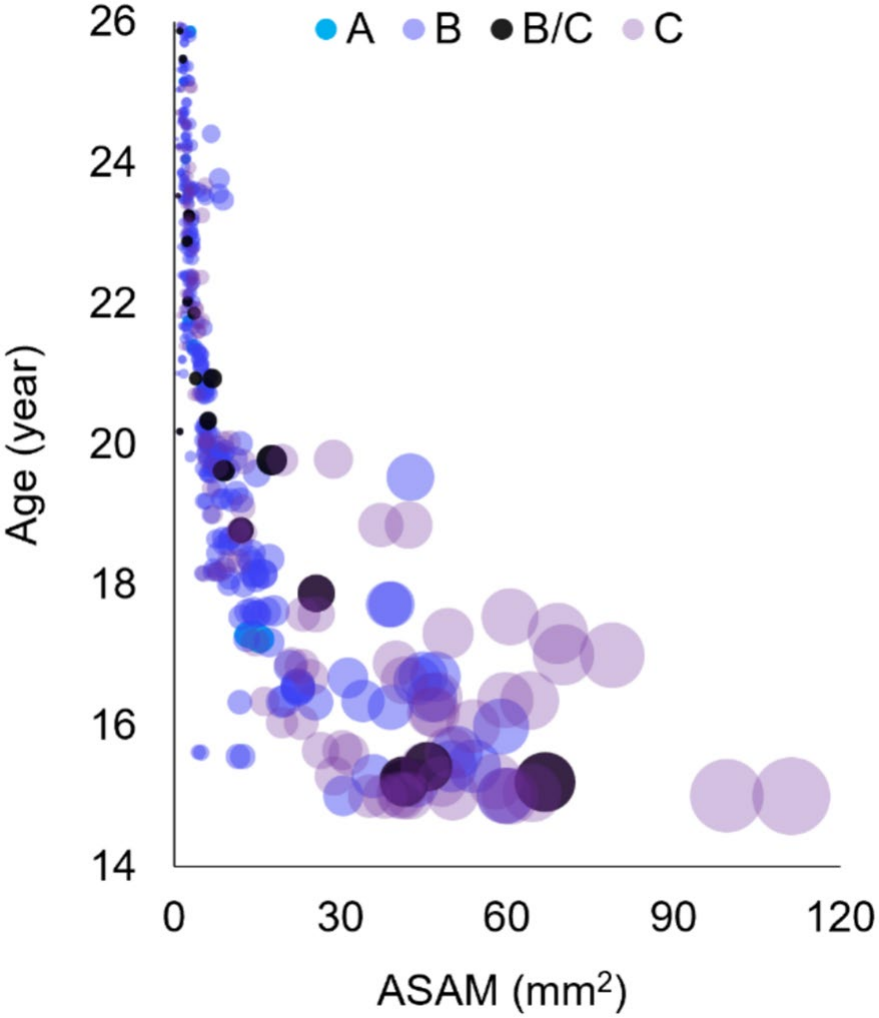
Relationship between chronological age and ASAM of IMTM. Each bubble represents an MTM, with the bubble size corresponding to the ASAM, and the colour indicating the impaction level

ASAM values in the sample ranged from 0.44 to 111.24 mm^2^, with an overall mean of 11.53 ± 16.50 mm^2^ and a median of 4.86 mm^2^ (IQR: 2.35–11.92) (Table 3). Figure 3 shows a negative association between ASAM and age (*ρ* = −0.89, *p* < 0.001), with age explaining 79.2% of the variance in ASAM. No significant difference was found between the right and left IMTM (*p* = 0.53), therefore, the data were pooled for subsequent analyses.

**Table 3:** Mean and median ASAM (mm^2^) by age groups

Group	N (%)	Mean ± SD	Median (IQR)	95% C.I
15.0–15.9	38 (8.5)	45.64 ± 21.41	44.36 (34.28, 58.16)	[38.60, 52.68]
16.0–16.9	29 (6.5)	32.63 ± 13.88	25.39 (21.69, 45.66)	[27.35, 37.91]
17.0–17.9	24 (5.4)	29.06 ± 21.00	17.54 (14.46, 39.03)	[20.20, 37.93]
18.0–18.9	37 (8.3)	12.39 ± 7.32	11.57 (8.55, 14.00)	[9.95, 14.83]
19.0–19.9	53 (11.9)	9.56 ± 6.26	7.89 (6.68, 9.49)	[7.83, 11.28]
20.0–20.9	36 (8.1)	5.73 ± 1.90	5.70 (5.14, 6.14)	[5.09, 6.37]
21.0–21.9	46 (10.3)	3.27 ± 1.30	3.47 (2.06, 4.46)	[2.89, 3.66]
22.0–22.9	45 (10.1)	2.74 ± 0.69	2.60 (2.35, 3.31)	[2.53, 2.95]
23.0–23.9	62 (13.9)	2.87 ± 1.61	2.56 (1.97, 3.15)	[2.46, 3.27]
24.0–24.9	41 (9.2)	1.88 ± 0.97	1.97 (1.35, 2.21)	[1.57, 2.19]
25.0–25.9	35 (7.8)	1.84 ± 0.65	1.70 (1.39, 2.21)	[1.62, 2.07]

*%* percentage, *IQR* interquartile range, *C.I* confidence interval

### Impaction level

**Fig. 4:**
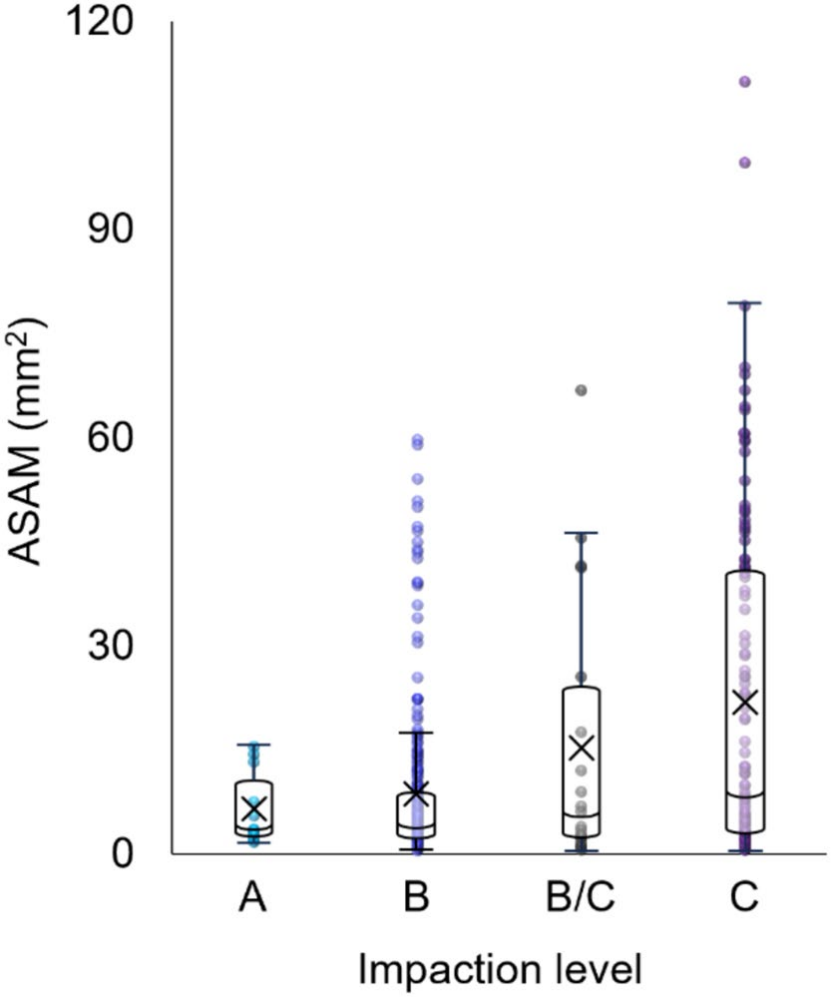
Boxplots of ASAM distribution by impaction level, showing an increasing trend in the mean (X), median, and variability with greater impaction depth

Table 4 presents the distribution, mean, and median ASAM across impaction levels. Levels B (67.3%) and C (25.3%) comprised the majority of the sample, while level A accounted for the fewest cases (2.9%). Both the mean and median ASAM, as well as variability, increased with greater impaction depth, as illustrated in Fig. 4.

**Table 4:** Distribution of IMTM, mean, and median ASAM (mm^2^) by impaction level

Level	N (%)	Mean ± SD	Median (IQR)	95% C.I
A	13 (2.9)	6.01 ± 5.03	3.40 (2.52, 10.40)	[2.97, 9.05]
B	300 (67.3)	7.79 ± 10.47	3.63 (2.14, 8.24)	[6.60, 8.98]
B/C	20 (4.5)	14.70 ± 19.16	5.01 (2.34, 23.60)	[5.73, 23.67]
C	113 (25.3)	21.53 ± 24.11	8.80 (2.95, 40.24)	[17.03, 26.02]

ASAM was strongly and negatively correlated with age across all four levels, with correlation coefficients ranging from *ρ* = −0.86 to −0.94 (*p* < 0.001). No specific trend was observed in the strength of this correlation (Table 5).

**Table 5:** Spearman’s rho correlation with age by impaction level

Level	Spearman ρ	95% C.I	Variance explained (%)
A	-0.94	[−1.00, −0.83]	88
B	-0.86	[−1.00, −0.84]	74
B/C	-0.88	[−1.00, −0.75]	77
C	-0.90	[−1.00, −0.87]	81

Correlations for all levels were significant (*p* < 0.001)

Levene’s test indicated a violation of homogeneity of variances (*F*(3, 442) = 53.65, *p* < 0.001). Welch’s ANOVA showed significant differences in ASAM between impaction levels (*F*(3, 41.74) = 12.80, *p* < 0.001), with a moderate effect size (*ω*^2^ = 0.13). Post hoc Games-Howell comparisons indicated that level C impaction had significantly larger ASAM than levels A and B (*p* < 0.001), while other pairwise comparisons were not significant (Table 6).

**Table 6:** Pairwise comparisons of ASAM between impaction levels

Comparison	Mean difference	95% C.I	p-value
A vs B	−1.79	[−6.11, 2.54]	0.65
A vs B/C	−8.70	[−21.17, 3.78]	0.24
A vs C*	−15.52	[−22.49, −8.55]	< 0.001
B vs B/C	−6.91	[−19.03, 5.21]	0.40
B vs C*	−13.74	[−19.84, −7.63]	< 0.001
B/C vs C	−6.83	[−19.99, 6.34]	0.50

^*^Significant at *p* < 0.05

## Discussion

The apical maturity of MTM typically occurs between 18 and 19 years of age [12], but may be delayed until 20 to 23 years [9, 13, 14]. Asif et al. [8, 9] used a 3D approach to measure the apical surface area of MTM and reported a correlation with chronological age. The present study applied the same approach in IMTM to examine this correlation through ASAM, and to compare differences between impaction levels. Developing teeth are typically described as ‘unerupted’ but may also be classified as ‘impacted’ if eruption is hindered by local or systemic factors and the average age of alveolar emergence has passed [15]. In this study, all MTM meeting these criteria were categorised as impacted.

The inverse correlation observed between IMTM apical surface area and age is consistent with previous findings in impacted and non-impacted MTM [9], maxillary canines [16], and studies based on 2D imaging [13, 14, 17]. These results confirm the developmental pattern in which apical surface area decreases as root formation progresses. The decrease in surface area between ages 15 and 18 was the most notable, with the steepest decline between 17 and 18 years. These findings support the application of ASAM, regardless of impaction status, as an indicator of dental maturity for age estimation, especially in identifying and distinguishing individuals having attained the age of majority (18 years) in the Malaysian population [4].

Despite similarities in the general pattern, apical narrowing in IMTM was relatively more gradual than in ungrouped MTM [9]. The direct relationship observed between impaction depth and apical foramen measurement corroborates earlier reports of developmental delay in IMTM [5, 18]. The present study further demonstrated that, although the difference was subtle, the delay at level C was particularly significant, as same-age comparisons across levels, together with the effect size, provided credible support for this observation. Friedrich et al. [19] proposed that such delays are more likely attributable to physical factors, such as limited eruption space or mechanical obstruction, rather than pathological causes. This reflects the broader physiology of tooth eruption, in which root development is closely associated with eruption and may be halted or slowed when the path is obstructed. Interestingly, despite this delay, the strength of the correlation between ASAM and age remained consistent across impaction depths. This seemingly paradoxical finding presents a new challenge, particularly in estimating age for level C MTM, as current methods may yield misleading results with wider margins of error.

Previous studies on IMTM have reported population differences in the distribution of impaction levels [20–22]. In the present study, which included Malaysian nationals from the west coast of Peninsular Malaysia, level B was the most frequent impaction, which contrasts with findings from a northeastern Malaysian population, where level A was most common, followed by B and C [22]. Another pattern among Asian-Indian groups was reported with a more even distribution across levels B (39.0%), C (34.0%), and A (27.0%) [21]. Such variations may, in part, be explained by age differences between study samples, as younger individuals are less likely to exhibit level A impactions due to incomplete root development, whereas older samples, such as those in the northeastern study, are more likely to do so. Biological variation among populations and potential misclassification due to inconsistent classification criteria may also have contributed. To minimise such inconsistencies and improve repeatability, the modified level B/C category was introduced.

One key implication of comparing disproportionate samples is the potential for bias, as smaller sample sizes may inadequately represent the population [23]. In the present study, this concern was partly mitigated by the comparable and consistent ages across the four groups. While standardising sample sizes across levels could allow for more balanced and comprehensive analyses, non-parametric tests remain appropriate when the assumption of normality is not met. In addition, this limitation was addressed through the use of effect size, which reflects the magnitude and practical significance of the findings, particularly the ASAM difference observed at level C.

Another limitation of this study was the exclusion of other impaction parameters such as angulation [24] and ramus position [10]. Other factors, including sex and root number, were also not considered. While some studies suggest these may influence development, their effects appear minimal when assessed independently [6]. Their combined impact remains unclear; however, future studies using multivariate approaches with these parameters could provide a more robust understanding of their potential interactions and overall implications for dental age estimation.

In conclusion, this study demonstrated a clear inverse correlation between the apical surface area of IMTM and chronological age, with the most marked reduction occurring around 18 years. A significant delay was evident only in level C impactions, and IMTM can therefore serve as reliable indicators of maturity for age estimation, particularly in identifying individuals at or above 18 years of age. However, caution is required when assessing level C impactions due to their delayed development and the potential for increased estimation error.

## Supplementary Information

Below is the link to the electronic supplementary material.

Supplementary file1 (XLSX 83 KB)

## Data Availability

Data were recorded in Microsoft Excel (Version 18, Microsoft Corporation) and are provided in Online Resource 1. Patient IDs were anonymised, and CBCT images were excluded to maintain confidentiality.
